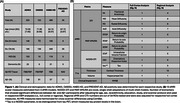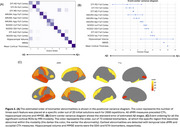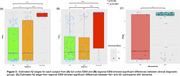# Event‐Based Modeling Reveals the Order of Microstructural and Macrostructural Changes Along the Amyloid Positivity Continuum

**DOI:** 10.1002/alz70856_105352

**Published:** 2026-01-08

**Authors:** Shayan Javid, Alyssa H Zhu, Sunanda Somu, Kevin Low, Siddharth Narula, Julio E Villalon‐Reina, Emma J Gleave, Sophia I Thomopoulos, Paul M. Thompson, Neda Jahanshad, Talia M Nir

**Affiliations:** ^1^ Imaging Genetics Center, Mark and Mary Stevens Neuroimaging & Informatics Institute, University of Southern California, Marina del Rey, CA, USA

## Abstract

**Background:**

Diffusion MRI (dMRI) is sensitive to microstructural brain abnormalities, which may precede standard MRI macrostructural changes in the progression of Alzheimer's disease (AD) and related dementias. dMRI may allow for earlier amyloid beta (Aβ) detection and intervention before significant cognitive decline and help differentiate between Aβ+ and Aβ‐ dementias. Event‐based modeling (EBM) is a probabilistic data‐driven method applicable to cross‐sectional data that can order multimodal biomarkers as they become abnormal in the course of disease progression. We used EBM to examine whether changes in cortical microstructure precede T1‐weighted (T1w) cortical thickness (CTh) and hippocampal volume changes with respect to Aβ status.

**Method:**

T1w, dMRI, and Aβ‐PET data were analyzed for 1,033 participants from ADNI3, HABS‐HD, OASIS3, and PREVENT‐AD (age range: 46‐92; Figure 1A). DTI and two more advanced models were fitted to the dMRI data, yielding 10 diffusion metrics extracted from the cortical gray matter (Figure 1B). CTh and hippocampal volumes were also extracted. All MRI measures were harmonized for cross‐scanner differences using ComBat. First, we identified whether full cortex dMRI measures, total CTh, hippocampal volume, and MMSE differed between Aβ‐ and Aβ+ individuals. Measures that differed significantly were then ordered based on the Aβ‐ to Aβ+ continuum using EBM. To validate the resulting order, we tested for associations between EBM‐derived subject‐wise Aβ stage estimates and 1) clinical diagnosis and 2) Aβ positivity within the dementia group indicative of AD.

**Result:**

All biomarkers differed significantly between Aβ+ and Aβ‐ participants and were sequenced with EBM (Figure 2A). Full cortex changes in all dMRI measures preceded hippocampal volume, MMSE, and total CTh. Subject‐level Aβ stage estimates showed significant differences between all diagnostic groups (Figure 3A). In regional analyses, 73 biomarkers were associated with Aβ status (Figure 1B). Regional EBM revealed earliest abnormalities in pericalcarine CTh and temporal dMRI measures (Figure 2C). Subject‐level stages showed significant differences between diagnostic groups (Figure 3B), and between Aβ‐ and Aβ+ dementia participants (Figure 3C).

**Conclusion:**

Aβ‐related abnormalities in dMRI cortical microstructure may precede abnormalities in traditional biomarkers of brain atrophy in AD. Future work will determine if EBM‐derived Aβ stage estimates may also help to differentiate AD from other dementia subtypes.